# Inhibitory effects of herbal monomers on ferroptosis in renal fibrosis: a review and mechanistic study

**DOI:** 10.3389/fphar.2025.1610573

**Published:** 2025-07-22

**Authors:** Kaixiang Liu, Min Yu, Yangyang He, Ting Wang, Guisen Li, Li Wang, Xiang Zhong

**Affiliations:** Department of Nephrology and Institute of Nephrology, Sichuan Provincial People’s Hospital, School of Medicine, University of Electronic Science and Technology of China, Chengdu, China

**Keywords:** Chinese herbal medicine, ferroptosis, chronic kidney disease, renal fibrosis, monomers

## Abstract

**Background and purpose:**

Renal fibrosis is a common characteristic of chronic kidney disease (CKD). Studies have confirmed the role of ferroptosis in the pathogenesis of various kidney diseases, making it a new research hotspot in the field of renal fibrosis. Monomers of Chinese herbal medicines (CHMs) can improve renal fibrosis by multi-target inhibition of ferroptosis. This review aimed to explore the roles and mechanisms of CHMs in renal fibrosis.

**Methods:**

Using the keywords “ferroptosis”, “chronic kidney disease”, “renal fibrosis”, “Chinese herbal medicine”, “natural products”, “bioactive components”, and “herb”, we conducted an extensive literature search of several databases, including PubMed, Web of Science, CNKI, and Wanfang database, to identify studies reporting the role of CHM monomers in inhibiting ferroptosis and improving renal fibrosis. The names of the plants covered in the review have been checked through MPNS (http://mpns.kew.org). All monomers of CHMs were identified in the Pharmacopoeia of the People’s Republic of China.

**Results:**

In total, 21 monomers of CHMs were identified in this study, most of which were flavonoids, followed by terpenoids and coumarins. This review showed that monomers of CHMs inhibited ferroptosis and improved renal fibrosis through multi-target mechanisms. They maintained iron homeostasis by acting on NCOA4 and Nrf2 to reduce ferritinophagy. They also inhibited lipid peroxidation and regulated the antioxidant system by modulating ACSL4, NOX4, Nrf2, FSP1, and GPX4 and inhibiting Smad3 to improve renal fibrosis.

**Conclusion:**

Monomers of CHMs effectively inhibited ferroptosis and prevented renal fibrosis in various animal models and cell models of CKD. However, further in-depth studies with better designs are needed to identify the exact targets of monomers of CHMs and improve the treatment of renal fibrosis and CKD.

## 1 Introduction

Chronic kidney disease (CKD) is a major global health concern, affecting 8%–16% of the global population (Chen et al., 2019). In 2022, CKD was estimated to affect ∼850 million people globally (Kovesdy, 2011; Romagnani et al., 2025). The primary risk factors for CKD include glomerular diseases, diabetes, hypertension, obesity, and aging (Sui et al., 2020). CKD can advance to end-stage kidney disease (ESKD), necessitating dialysis or kidney transplantation. By 2030, an estimated 5 million patients will need kidney replacement therapy worldwide, doubling the 2010 figures (Liyanage et al., 2015). The number of patients needing dialysis in China is rising rapidly, with 207,863 new cases in 2023, increasing the total number to 1 million (Webster et al., 2017). These statistics highlight the growing burden of CKD and the pressing need for effective treatments. Renal fibrosis is a common characteristic of CKD. It involves excessive deposition of extracellular matrix (ECM) in the interstitial space, disrupting normal kidney architecture and contributing to renal failure. Inhibiting the progression of renal fibrosis is essential for preserving renal function (Huang et al., 2023). However, effective treatments to decelerate the progression of CKD remain limited, and disease progression can be rarely reversed (Li S. et al., 2024).

Ferroptosis is an iron-dependent form of programmed cell death, primarily characterized by the accumulation of intracellular lipid peroxides (Dixon et al., 2012). This form of cell death is strongly associated with iron overload, lipid peroxidation, and impaired antioxidant capacity (Zhang et al., 2022; Zhou L. et al., 2022). Previous studies have demonstrated a strong correlation between ferroptosis and kidney disease, suggesting that regulation of ferroptosis can serve as a novel therapeutic strategy for the treatment of kidney diseases (Li S. et al., 2024; Lai et al., 2024; Wang F. et al., 2024). Targeting ferroptosis with specific inhibitors, such as ferrostatin-1 (Fer-1) and liproxstatin-1 (Lip-1), promotes adaptive cell repair and mitigates fibrosis, highlighting ferroptosis inhibition as a promising strategy for mitigating renal fibrosis (Zhou L. et al., 2022; Balzer et al., 2022; Wang J. et al., 2022). However, the long-term off-target consequences of these inhibitors, particularly drug interactions and cumulative organ-specific toxicities, need further systematic assessments (Conrad et al., 2021). These concerns underscore the critical need for ferroptosis inhibitors with improved efficacy and optimized safety profiles.

The discovery of artemisinin for the treatment of malaria indicates that monomers of Chinese herbal medicines (CHMs) can effectively address complex health issues (Tu, 2011). Notably, monomers derived from CHMs exhibit superior biocompatibility and multi-target regulatory capacity compared to synthetic compounds, making them promising candidates for pharmacological intervention (Parkash et al., 2018). CHMs have long been used in clinical practice, and recent pharmacological studies have elucidated their nephroprotective mechanisms in CKD through multi-target modulation of ferroptosis and renal fibrosis (Gong et al., 2024). The main monomers of CHMs include flavonoid glycosides, triterpenoid derivatives, coumarin analogs, isoquinoline alkaloids, steroidal saponins, polyphenolic acids, and enzymatic cofactors. Previous studies have shown that CHM-derived monomers and their compounds exhibit significant therapeutic efficacy in treating various diseases while maintaining low toxicity and minimal side effects, making them invaluable candidates for modern drug development (Huang et al., 2024; Singh et al., 2024).

This review provides a comprehensive overview of monomer research methods and identifies key targets for regulating ferroptosis. It also discusses the effects and mechanisms of these targets on renal fibrosis, establishing a foundation for future studies on how monomers can enhance ferroptosis and mitigate renal fibrosis.

## 2 CKD and renal fibrosis

Renal fibrosis is a hallmark feature of the progression of CKD to ESKD, characterized by tubular atrophy, chronic interstitial inflammation, fibrosis, glomerulosclerosis, and vascular rarefaction (Yuan et al., 2022). Renal fibrosis can be caused by various conditions affecting the kidneys, including glomerular diseases, ischemia-reperfusion injury, diabetic nephropathy, and nephrotoxic agents (Han et al., 2024). Its severity is positively correlated with decreased renal function (Rayego-Mateos and Valdivielso, 2020; Risdon et al., 1968; Rodríguez-Iturbe et al., 2005). Renal fibrosis is a multifaceted pathological process driven by interactions between various renal cell types and multiple molecular pathways, such as the TGF-β/Smads, Wnt/β-catenin, and NF-κB pathways (Gu et al., 2024; Tan et al., 2014; Niu et al., 2025). This process is characterized by several pathological alterations, including ECM deposition, epithelial-mesenchymal transition (EMT) of renal tubular cells, fibroblast activation, immune cell infiltration, and renal cell apoptosis (Rayego-Mateos and Valdivielso, 2020). Transforming growth factor-β1 (TGF-β1) is a main mediator of renal fibrosis, promoting ECM deposition by stimulating fibroblast proliferation and enhancing collagen synthesis (Lan, 2011). TGF-β activates Smad2/3 through TGF-β receptor I (TGF-βRI) and TGF-β receptor II (TGF-βRII). Activated Smad2/3 forms a complex with Smad4 and translocates to the nucleus, where it modulates the expression of genes associated with EMT of renal tubular epithelial cells (TEC) (Lovisa et al., 2015). Via EMT, epithelial cells can excessively produce collagen I (Col-I) and fibronectin (FN), thereby driving renal interstitial fibrosis (Xing et al., 2022; Yuan et al., 2019). Moreover, pathological conditions, such as renal ischemia, hypoxia, renal tubular epithelial cell injury, diabetes, and hypertension, exacerbate renal fibrosis (Naas et al., 2023; Nogueira et al., 2017; Qiu et al., 2023; Zhang et al., 2020).

## 3 Ferroptosis in renal fibrosis

### 3.1 Core mechanisms of ferroptosis

Ferroptosis is a novel form of cell death that differs morphologically and biochemically from apoptosis, necrosis, and autophagy. It was first identified by Dixon et al., in 2012 (Dixon et al., 2012). Ferroptosis is an iron-dependent lipid peroxidation mechanism, which plays a crucial role in cell survival and homeostasis (Liu et al., 2022; Jiang et al., 2021; Li J. et al., 2020; Ru et al., 2024). It is currently believed that ferroptosis is an important target for developing new therapeutic strategies (Stockwell, 2022).

#### 3.1.1 Iron metabolism

**FIGURE 1:**
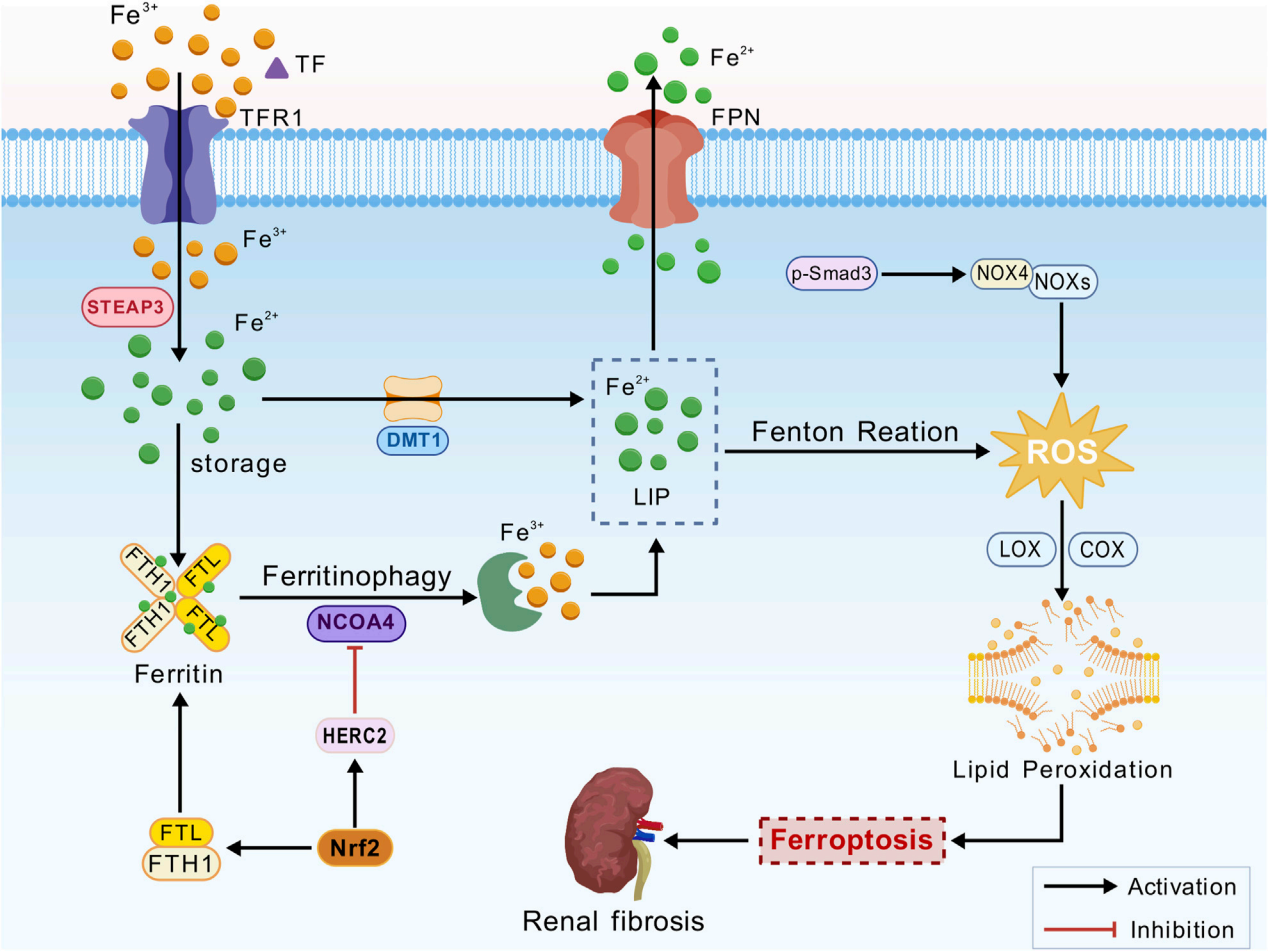
Iron metabolism mechanism. Transferrin (TF), transferrin receptor 1 (TFR1), ferroportin 1 (FPN1), six-transmembrane epithelial antigen of the prostate 3 (STEAP3), divalent metal transporter 1 (DMT1), labile iron pool (LIP), reactive oxygen species (ROS), ferritin heavy chain 1 (FTH1), ferritin light chain (FTL), nuclear receptor coactivator 4 (NCOA4), nuclear factor erythroid 2-related factor 2 (Nrf2), NADPH oxidases (NOXs), lipoxygenase (LOX), cyclooxygenase (COX), polyunsaturated fatty acids (PUFAs), and phosphorylated Smad3 (p-Smad3).

Ferroptosis is an iron-dependent form of cell death closely associated with impaired iron metabolism (Figure 1). Extracellular ferric iron (Fe^3+^) is internalized via transferrin (TF), which binds to transferrin receptor 1 (TFR1). Iron export is primarily mediated by ferroportin 1 (FPN1). Six-transmembrane epithelial antigen of the prostate 3 (STEAP3) reduces Fe^3+^ to ferrous iron (Fe^2+^) in endosomes. Divalent metal transporter 1 (DMT1) facilitates the release of Fe^2+^ from endosomes into the cytoplasmic labile iron pool (LIP). Free Fe^2+^ in the LIP participates in the Fenton reaction (Fe^2+^ + H_2_O_2_ → Fe^3+^ + ·OH + OH^−^), generating reactive oxygen species (ROS), particularly hydroxyl radicals. In pathological conditions, an imbalance between FPN1 and DMT1, such as FPN1 downregulation or DMT1 overexpression, leads to intracellular iron overload (Feng et al., 2020). Intracellular iron is stored in the form of ferritin, a complex with two major subunits: ferritin heavy chain 1 (FTH1) and ferritin light chain (FTL). FTH1 primarily maintains the structural integrity of the ferritin shell, while FTL is critically involved in iron storage and release. These subunits work synergistically to maintain intracellular iron homeostasis (Ru et al., 2024; Chen et al., 2020; Latunde-Dada, 2017). Nuclear receptor coactivator 4 (NCOA4) facilitates ferritin degradation through ferritinophagy, thereby leading to the release of Fe^2+^ and increasing the burden of labile LIP. Additionally, NCOA4 mediates FTH1 autophagy, thereby modulating sensitivity to ferroptosis (Luo et al., 2025; Yu et al., 2024).

Nuclear factor erythroid 2-related factor 2 (Nrf2) is a critical transcription factor that plays a significant role in the kidneys, particularly in the context of oxidative stress and inflammation (Dächert et al., 2020). Activation of Nrf2 promotes iron storage, reduces iron uptake by cells, and limits ROS production (Han et al., 2024). Nrf2 could regulate iron homeostasis by controlling HERC2, an E3 ubiquitin ligase for NCOA4 and FBXL5, as well as VAMP8, thereby facilitating autophagosome-lysosome fusion. Furthermore, NRF2 modulates the transcription of FTL and FTH1, ensuring the safe storage of intracellular iron and limiting the accumulation of free Fe^2+^. This mechanism mitigates lipid peroxidation and suppresses ferroptosis via the Fenton reaction (Anandhan et al., 2023; Cheng et al., 2023; Zhao et al., 2025). In the presence of iron overload, Fe^2+^ catalyzes ROS production via the Fenton reaction, generating highly reactive hydroxyl radicals (·OH). These radicals attack polyunsaturated fatty acids (PUFAs) in the cell membrane, initiating a lipid peroxidation chain reaction, membrane disruption, and ferroptosis (Bayir et al., 2023).

#### 3.1.2 Lipid peroxidation

**FIGURE 2:**
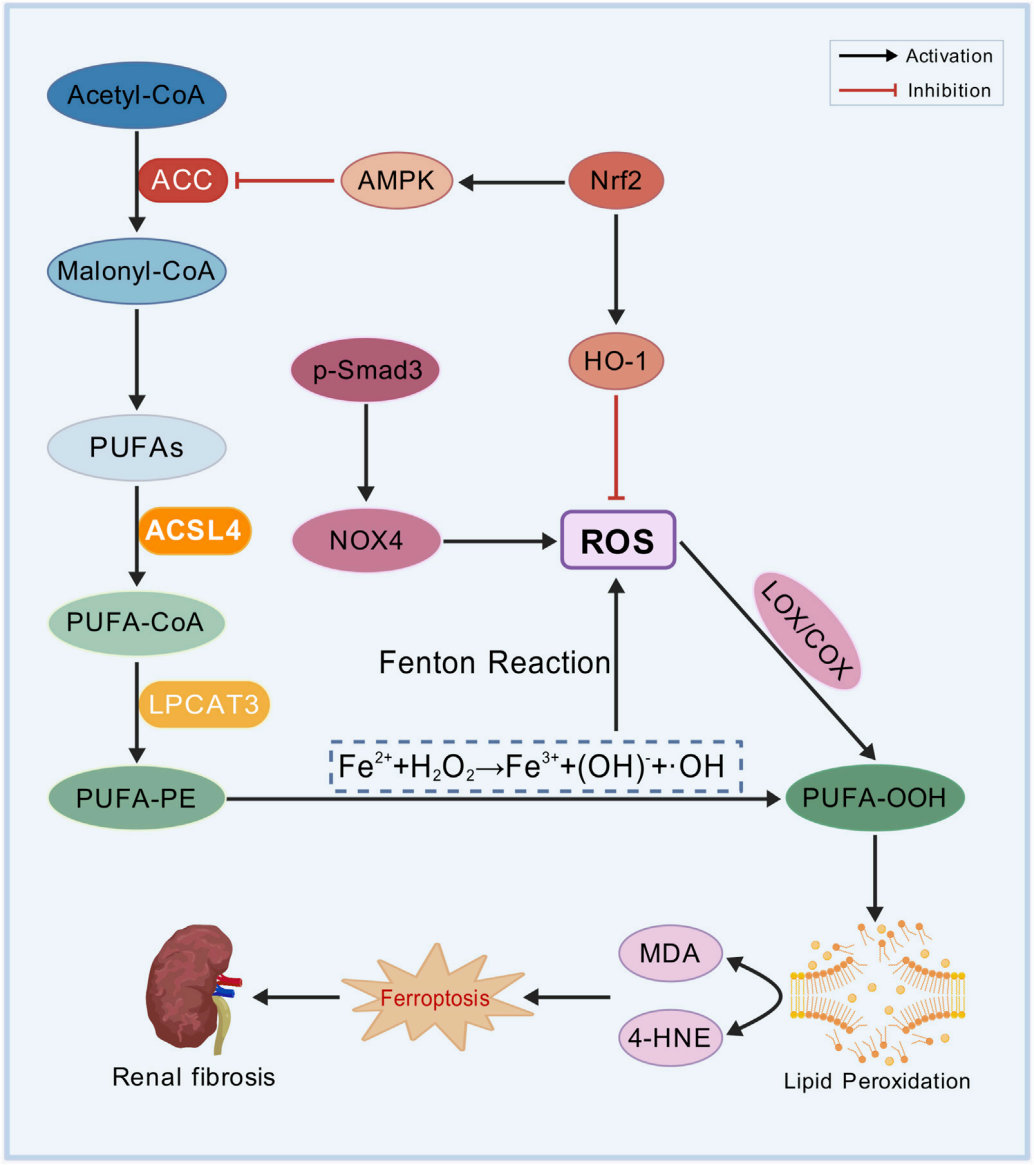
Lipid peroxidation mechanism. Polyunsaturated fatty acids (PUFAs), acetyl-CoA carboxylase (ACC), acyl-CoA synthetase long-chain family member 4 (ACSL4), arachidonic acid (AA), adrenic acid (AdA), phosphatidylethanolamine (PE), lysophosphatidylcholine acyltransferase 3 (LPCAT3), PUFA-phospholipids (PUFA-PE), NADPH oxidases (NOXs), reactive oxygen species (ROS), lipoxygenases (LOXs), cyclooxygenases (COXs), lipid hydroperoxides (PUFA-OOH), nuclear factor erythroid 2-related factor 2 (Nrf2), heme oxygenase-1 (HO-1), AMP-activated protein kinase (AMPK), malondialdehyde (MDA), and 4-hydroxynonenal (4-HNE).

Lipid peroxidation is one of the essential factors driving ferroptosis and serving as a major cause of cell death (Figure 2) (Rochette et al., 2022). The oxidative metabolism of PUFAs is the central mechanism of ferroptosis. Acetyl-CoA, the initial substrate for fatty acid synthesis, is catalyzed by acetyl-CoA carboxylase (ACC) to generate malonyl-CoA, providing precursors for lipid biosynthesis. Acyl-CoA synthetase long-chain family member 4 (ACSL4) facilitates the conversion of free PUFAs, such as arachidonic acid (AA) and adrenic acid (AdA), into PUFA-CoA. These PUFA-CoA molecules are subsequently incorporated into the sn-2 position of phosphatidylethanolamine (PE) by lysophosphatidylcholine acyltransferase 3 (LPCAT3), forming highly oxidation-sensitive PUFA-phospholipids (PUFA-PE), including AA-PE and AdA-PE. After the activation of NADPH oxidases (NOXs)-derived ROS, lipoxygenases (LOXs) and cyclooxygenases (COXs) directly oxidize PUFA-PE, leading to the production of lipid hydroperoxides (PUFA-OOH) (Lai et al., 2024; Lee et al., 2021).

NRF2 mitigates oxidative stress by activating antioxidant genes, such as heme oxygenase-1 (HO-1), to eliminate ROS. Additionally, NRF2 suppresses lipid peroxidation by inhibiting ACC via AMP-activated protein kinase (AMPK) (Lu et al., 2023). PUFA-OOH undergoes non-enzymatic degradation to generate malondialdehyde (MDA) and 4-hydroxynonenal (4-HNE), both of which can represent the severity of ferroptosis. 4-HNE and MDA compromise the integrity of cell membranes, leading to protein cross-linking, DNA damage, and mitochondrial dysfunction, finally inducing ferroptosis in renal TECs (Stockwell, 2022; Tang et al., 2021; Zhang X. et al., 2023).

#### 3.1.3 Antioxidant defense

**FIGURE 3:**
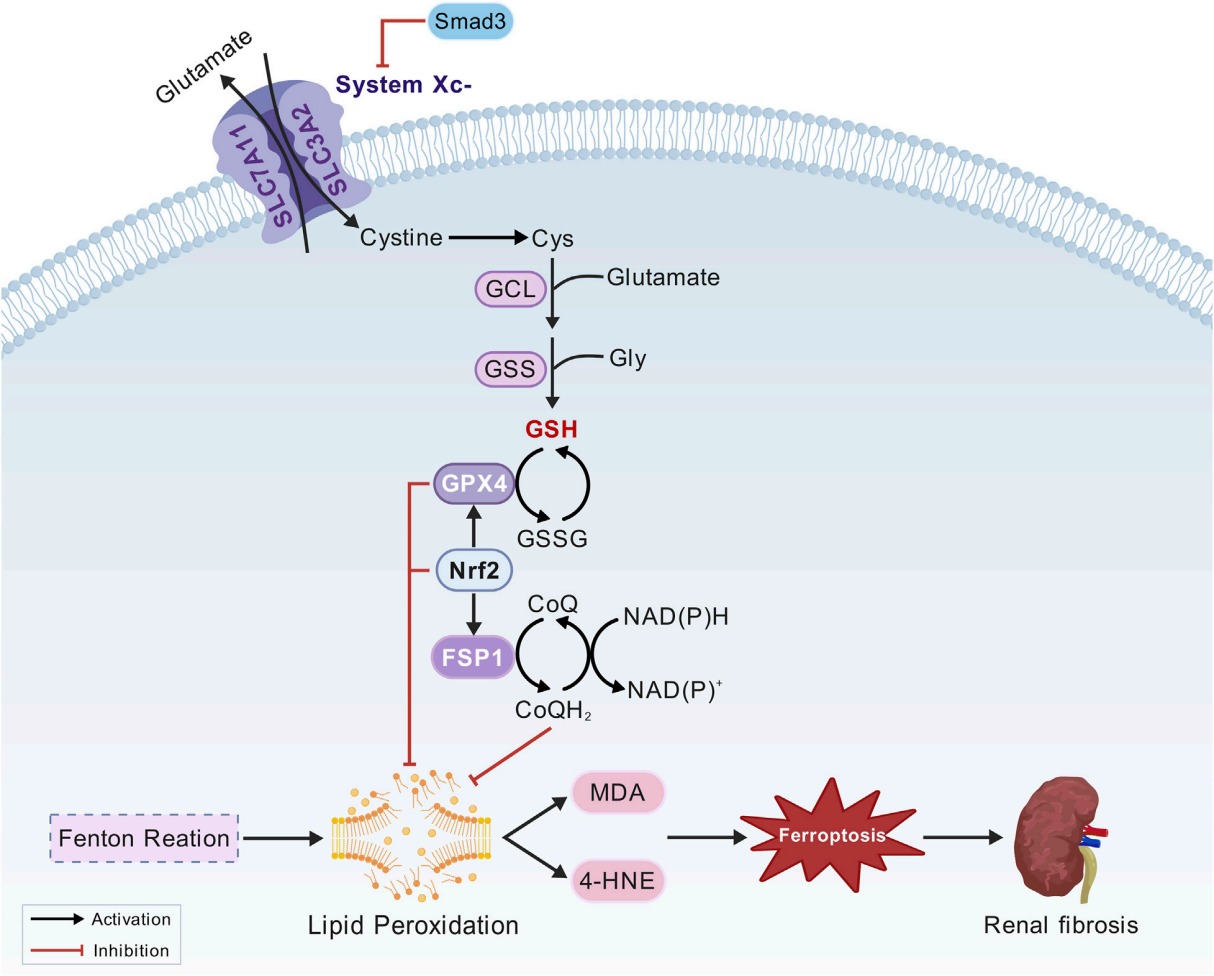
Oxidation system mechanism. Cystine/glutamate antiporter (System Xc^−^), glutathione peroxidase 4 (GPX4), solute carrier family 7 member 11 (SLC7A11), solute carrier family 3 member 2 (SLC3A2), glutathione (GSH), nuclear factor erythroid 2-related factor 2 (Nrf2), activating transcription factor 3 (ATF3), Mothers against decapentaplegic homolog 3 (Smad3), ferroptosis suppressor protein 1 (FSP1), coenzyme Q10 (CoQ10), ubiquinol (CoQ10H_2_), nicotinamide adenine dinucleotide phosphate (NADPH).

The core regulatory mechanisms of ferroptosis include three major antioxidant systems, namely the Xc^−^/GSH/GPX4 pathway, the FSP1/CoQ10 pathway, and the Nrf2/ARE pathway (Figure 3).

##### 3.1.3.1 The Xc-/GSH/GPX4 pathway

Inhibition of system Xc^−^ and glutathione peroxidase 4 (GPX4) expression leads to insufficient GSH synthesis, resulting in excessive lipid peroxidation and ROS accumulation in the intracellular space. This process plays a crucial role in triggering ferroptosis (Liu et al., 2022; Liu et al., 2023). The System Xc^−^ is composed of solute carrier family members solute carrier family 7 member 11(SLC7A11) and solute carrier family 3 member 2 (SLC3A2). It facilitates the transport of extracellular cystine into cells, where it is reduced to cysteine, a critical precursor for the synthesis of glutathione (GSH). As an antioxidant enzyme, GPX4 regulates ferroptosis and functions as a “scavenger” of lipid peroxides. It utilizes reduced glutathione to convert lipid hydroperoxides into their corresponding lipid alcohols, thereby mitigating lipid peroxidation and inhibiting ferroptosis. Therefore, GPX4 can regulate ferroptosis (Jia et al., 2020). Nrf2 can promote the expression of SLC7A11 and GPX4 under oxidative stress, thereby preventing ferroptosis (Dong et al., 2020; G et al., 2021). Activating transcription factor 3 (ATF3) induces ferroptosis by directly binding to the SLC7A11 promoter and suppressing its expression (Wang et al., 2020). Smad3, a central regulator of fibrosis, directly interacts with ATF3 and regulates its transcriptional activity (Shi et al., 2020).

##### 3.1.3.2 The FSP1/CoQ10 pathway

Using nicotinamide adenine dinucleotide phosphate (NADPH), ferroptosis suppressor protein 1 (FSP1) reduces coenzyme Q10 (CoQ10) to its active form, ubiquinol (CoQ10H_2_). As a lipophilic antioxidant, ubiquinol directly neutralizes lipid radicals, establishing a GPX4-independent defense mechanism against ferroptosis (Bersuker et al., 2019; Doll et al., 2019). The discovery of FSP1 unveiled the complex regulation of ferroptosis and offered new directions for targeted therapy.

##### 3.1.3.3 The Nrf2/ARE pathway

Nrf2 is a master regulator of cell defense systems involved in detoxification, antioxidant defense, and resolving inflammation and fibrosis (Murakami et al., 2023). Under oxidative stress, Nrf2 translocates to the nucleus and binds to the antioxidant response element (ARE), upregulating the expression of various antioxidant genes, including SLC7A11, GPX4, and HO-1. They are essential for reducing lipid hydroperoxides to their corresponding alcohols, thereby preventing the accumulation of lipid peroxides that trigger ferroptosis (Deng et al., 2020; Lu et al., 2021; Song and Long, 2020). By enhancing the expression of these protective factors, Nrf2 effectively prevents lipid peroxidation, thereby mitigating the oxidative damage in various pathological conditions, including neurodegenerative diseases, chronic inflammation, and cancer (Li et al., 2023; Xu et al., 2025). Nrf2 also plays a crucial role in regulating iron homeostasis by modulating the expression of HERC2 and VAMP8. The latter is involved in the fusion of autophagosomes and lysosomes. This regulation highlights the significance of Nrf2 inhibition in triggering ferroptosis (Anandhan et al., 2023). Furthermore, previous studies have shown that FSP1, a key inhibitor of ferroptosis, partly relies on Nrf2 for its expression. Targeting both FSP1 and Nrf2 may represent a promising therapeutic strategy for renal ferroptosis (Kim et al., 2023).

### 3.2 The involvement of ferroptosis in renal fibrosis

The kidney is a crucial organ involved in iron filtration and reabsorption, thereby regulating iron metabolism. Rich in mitochondria and exhibiting high oxygen uptake, the kidney is highly susceptible to oxidative stress. In patients with CKD, decreased expression of saturated fatty acids in the kidney impairs the transfer of iron from cells to the extracellular space, while overexpression of iron-transfer factors leads to iron deposition in renal tubules (Packer, 2024). Furthermore, the kidney has high lipoxygenase expression, making it susceptible to lipid peroxidation and ferroptosis. Previous studies have demonstrated that ferroptosis is closely associated with renal fibrosis subsequent to glomerulonephritis, renal ischemia-reperfusion injury, diabetic nephropathy, and kidney stones in animal models (Ru et al., 2024; Du et al., 2022; Zhou Y. et al., 2022).

#### 3.2.1 Renal tubular epithelial cell (TEC) injury induces ferroptosis-induced fibrosis

Accumulating evidence suggests that TECs plays diverse roles in renal repair or progression to CKD (Liu et al., 2018). TECs are involved in the kidney’s response to various harmful stimuli and also serve as the source of myofibroblasts (Kuppe et al., 2021). The activation of myofibroblasts and subsequent release of ECM are the core features of renal interstitial fibrosis. During renal fibrosis, TECs mediate the initial response to injury, initiating EMT, when myofibroblasts become the main effector cells (Hong et al., 2019).

TECs are rich in mitochondria and generate adenosine triphosphate (ATP) for cellular function through fatty acid oxidation (FAO) (Hwang and Chung, 2021). After renal TEC injury, mitochondrial dysfunction leads to the accumulation of mitochondrial ROS, which triggers ferroptosis (Su et al., 2023). Damaged TECs promote inflammation and fibrosis through several mechanisms, including EMT, the release of pro-inflammatory factors, and the activation of signaling pathways, such as the TGF-β1/Smad3 pathway (Liu et al., 2018). This process is accompanied by intracellular iron accumulation and lipid peroxide generation, leading to cell death and decreased kidney function (Linkermann et al., 2014). The proximal tubule (PT), being a key site for substance transport and reabsorption, has high energy demands and is particularly susceptible to oxidative damage. Recent findings suggest that PT cells exhibit a unique pro-inflammatory function after injury, which significantly attenuates the mechanisms inhibiting ferroptosis (Ide et al., 2021).

#### 3.2.2 Ferroptosis-inflammation-fibrosis

Ferroptosis not only directly affects the survival of renal TECs but also affects the inflammatory response by inducing macrophage polarization. Ferroptosis leads to macrophage polarization toward the M1 phenotype, releasing pro-inflammatory cytokines, such as TNF-α, IL-1β, and IL-6, which also aggravate renal inflammation and fibrosis. Studies have shown that macrophage polarization is closely associated with the severity of renal fibrosis, and inhibiting M1 polarization of macrophages can alleviate renal fibrosis (Tang et al., 2025).

**FIGURE 4:**
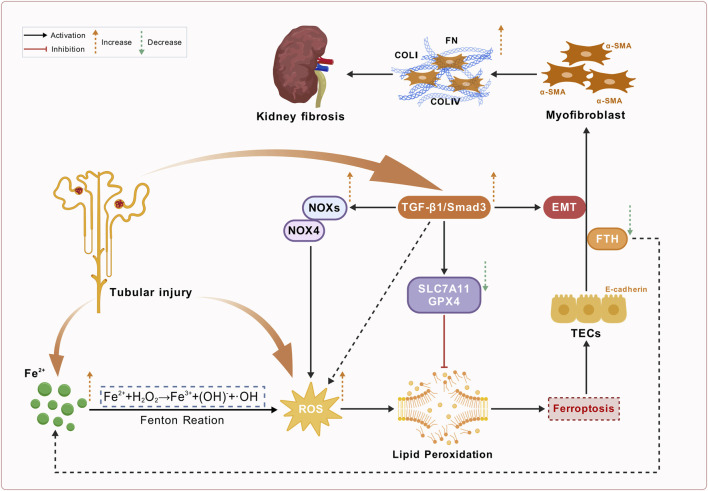
Schematic overview of renal fibrosis, ferroptosis, and TGF-β/Smad pathway activation in chronic kidney disease (CKD). This figure depicts the pathological mechanisms underlying renal fibrosis in CKD associated with ferroptosis. Tubular injury activates Fe^2+^ accumulation, leading to the Fenton reaction that generates reactive oxygen species (ROS). Increased ROS levels promote lipid peroxidation, which is inhibited by SLC7A11 and GPX4. Concurrently, the TGF-β1/Smad3 signaling, which is activated by tubular injury, enhances ROS production by triggering NOXs/NOX4 and promotes epithelial-mesenchymal transition (EMT). This complex pathway leads to myofibroblast activation through ferroptosis, characterized by α-SMA activation and enhanced production of ECM components (e.g., COLI, COLIV and FN), exacerbating kidney fibrosis.

Furthermore, ferroptosis promotes EMT by activating the TGF-β/Smad signaling pathway, leading to excessive deposition of extracellular matrix components, such as collagen. Studies have observed decreased levels of ferritin (FTH) during EMT. During EMT, ferritin releases free iron ions that induce iron overload, resulting in excessive ROS generation and ferroptosis (Zhou L. et al., 2022). This study supports the notion that iron overload contributes to renal fibrosis. These findings highlight the significant role of the ferroptosis-inflammation-fibrosis cascade in the pathogenesis of renal fibrosis. *In vivo* and *in vitro* studies have shown that ferroptosis inhibitors can inhibit fibrosis by downregulating the TGF-β1/SMAD3 signaling pathway, inhibiting the activation of fibroblasts, downregulating the expression of monocyte chemoattractant protein-1 (MCP-1), and inhibiting macrophage chemotaxis (Wang J. et al., 2022; Li et al., 2022; Ikeda et al., 2014; Lyu et al., 2025). Various forms of EMT activation, including TGF-β stimulation, increase the susceptibility to ferroptosis, thereby forming a vicious cycle (Lyu et al., 2025; Schwab et al., 2024) (Figure 4).

## 4 Monomers of CHMs protect against renal fibrosis by targeting ferroptosis

**FIGURE 5:**
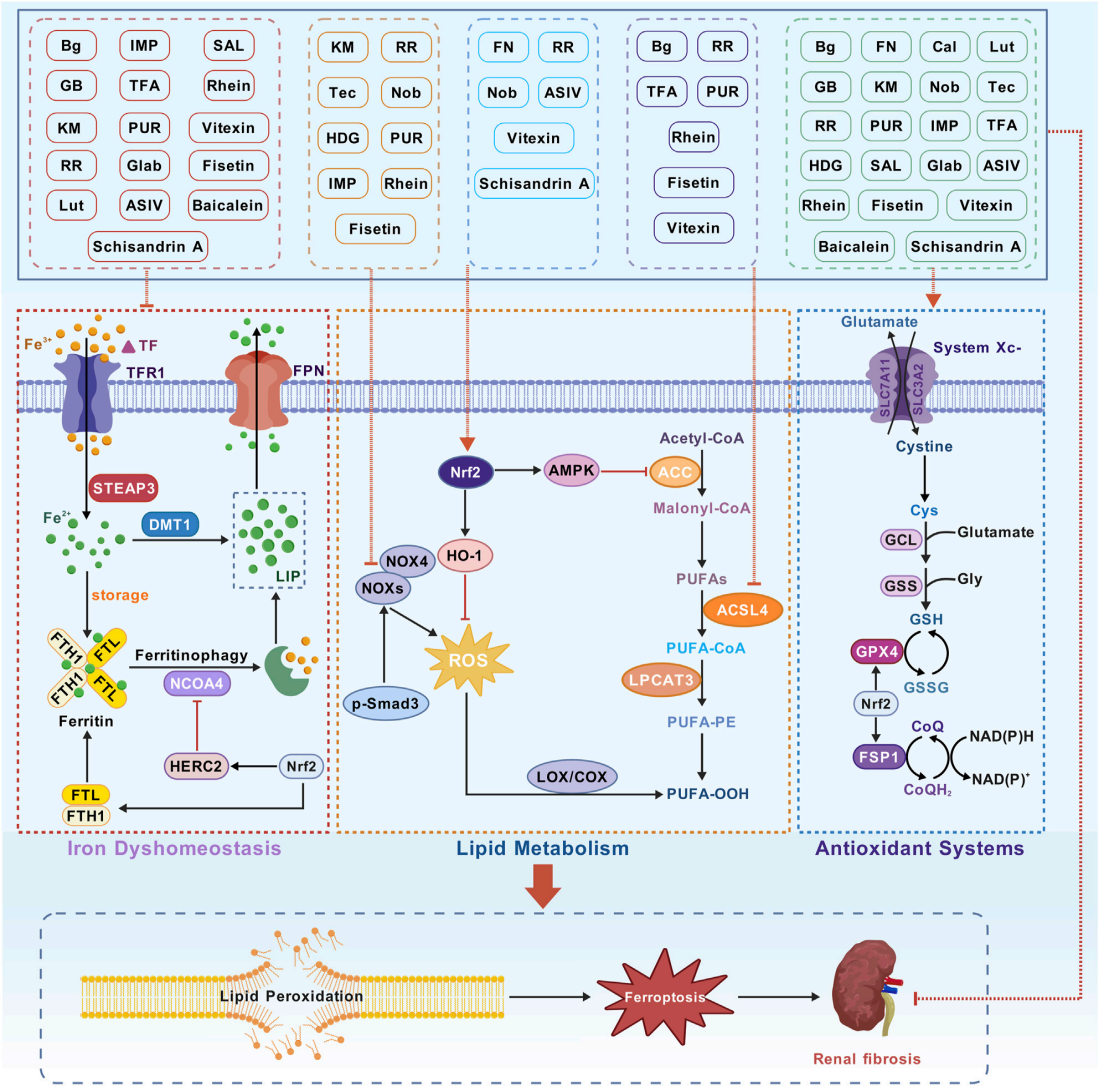
Monomer mechanism and target. Transferrin (TF), transferrin receptor 1 (TFR1), six-transmembrane epithelial antigen of the prostate 3 (STEAP3), divalent metal transporter 1 (DMT1), labile iron pool (LIP), ferritin heavy chain 1 (FTH1), ferritin light chain (FTL), nuclear receptor coactivator 4 (NCOA4), Polyunsaturated fatty acids (PUFAs), acetyl-CoA carboxylase (ACC), acyl-CoA synthetase long-chain family member 4 (ACSL4), lysophosphatidylcholine acyltransferase 3 (LPCAT3), PUFA-phospholipids (PUFA-PE), lipid hydroperoxides (PUFA-OOH), Cystine/glutamate antiporter (System Xc^−^), glutathione peroxidase 4 (GPX4), solute carrier family 7 member 11 (SLC7A11), solute carrier family 3 member 2 (SLC3A2), glutathione (GSH), nuclear factor erythroid 2-related factor 2 (Nrf2) and ferroptosis suppressor protein 1 (FSP1).

CHMs have been administered for more than 2000 years. A huge body of evidence suggests that CHMs monomers have great potential for treating renal fibrosis (Song et al., 2024). In this study, we conducted an extensive literature search in PubMed, Web of Science, CNKI, and Wanfang databases using keywords “ferroptosis”, “chronic kidney disease”, “renal fibrosis”, “Chinese herbal medicine”, “natural products”, “bioactive components”, and “herb” to identify studies reporting the role of CHM monomers in inhibiting ferroptosis and improving renal fibrosis. In total, 21 monomers and their roles, mechanisms, and targets in inhibiting ferroptosis and mitigating renal fibrosis were summarized (Table 1; Table 2; Table 3). The mechanisms and molecular targets of these monomers in inhibiting ferroptosis and alleviating renal fibrosis are summarized in Figure 5.

**TABLE 1:** The main monomers of Chinese herbal medicines for renal fibrosis treated.

Name	Molecular formula	Structure	Origin (scientific name)	Verified by the Chinese pharmacopoeia (2020)	Verified in MPNs	References
Fisetin	C15H10O6	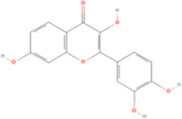	Sophora flavescens (kushen)	P211	Sophora flavescens Aiton	Wang et al. (2024b)
Imperatorin	C16H14O_4_	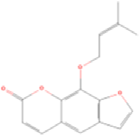	Angelica dahurica (baizhi)	P109	Angelica dahurica (Hoffm.) Benth. & Hook.f. ex Franch. & Sav.	Yang et al. (2024)
Tectorigenin	C16H12O_6_	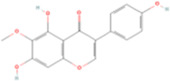	Belamcanda chinensis (shegan)	P297	Iris domestica (L.) Goldblatt & Mabb.	Li et al. (2022)
Formononetin	C16H12O_4_	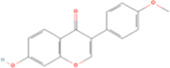	Astragalus membranaceus (huangqi)	P315	Astragalus mongholicus Bunge	Zhu et al. (2023)
Kaempferitrin	C27H_30_O_14_	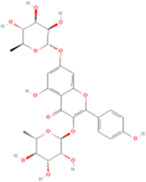	Astragalus membranaceus (huangqi)	P315	Astragalus mongholicus Bunge	Li et al. (2024b)
Vitexin	C21H_20_O_10_	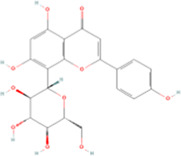	Nelumbo nucifera (lianzixin)	P285	Nelumbo nucifera Gaertn.	Song et al. (2023)
Bergapten	C_12_H_8_O_4_	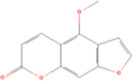	Citrus medica var. sarcodactylis (foshou)	P185	Citrus × limon (L.) Osbeck	Li et al. (2025)
Nobiletin	C21H_22_O_8_	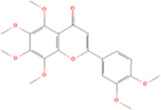	Citrus reticulata (chenpi)	P199	Citrus reticulata Blanco	Lo et al. (2022)
Baicalein	C15H_10_O_5_	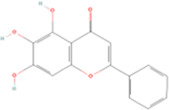	Scutellaria baicalensis (huangqin)	P314	Scutellaria baicalensis Georgi	Liang et al. (2024a)
Luteolin	C15H_10_O_6_	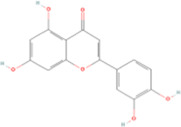	Lonicera japonica (jinyinhua)	P230	Lonicera japonica Thunb.	Ye et al. (2025)
Astragaloside IV	C41H_68_O_14_	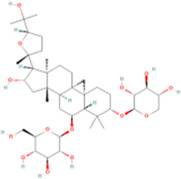	Astragalus membranaceus (huangqi)	P315	Astragalus mongholicus Bunge	Qin et al. (2022)
Ginkgolide B	C20H_24_O_10_	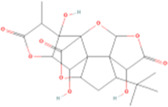	Ginkgo biloba (yinxingye)	P329	Ginkgo biloba L.	Chen et al. (2022)
Glabridin	C20H_20_O_4_	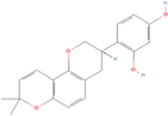	Glycyrrhiza uralensis (gancao)	P88	Glycyrrhiza glabra L.	Tan et al. (2022)
Hederagenin	C30H_48_O_4_	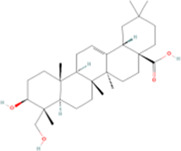	Acanthopanax senticosus (ciwujia)	P215	Eleutherococcus senticosus (Rupr. & Maxim.) Maxim.	Jia et al. (2024)
Puerarin	C21H_20_O_9_	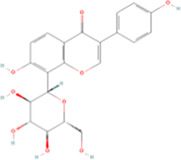	Pueraria lobata (gegen)	P347	Pueraria montana var. lobata (Willd.) Maesen & S.M.Almeida ex Sanjappa & Predeep	Hou et al. (2024)
Calycosin	C16H_12_O_5_	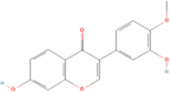	Astragalus membranaceus (huangqi)	P315	Astragalus mongholicus Bunge	Huang et al. (2022)
Rhein	C15H_8_O_6_	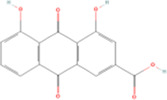	Rheum palmatum (dahuang)	P24	Rheum officinale Baill.	Xiong et al. (2023)
Total flavones of Abelmoschus (Quercetin)	C15H_10_O_7_	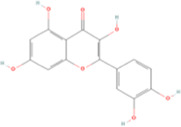	Abelmoschus manihot (huangshukuihua)	P319	Abelmoschus manihot (L.) Medik.	Wang et al. (2023)
Schisandrin A	C24H_32_O_6_	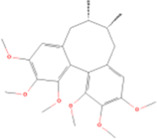	Schisandra chinensis (wuweizi)	P68	Schisandra chinensis (Turcz.) Baill.	Wang et al. (2022b)
Radish red	C33H_41_O_20_^+^	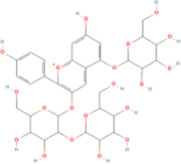	Raphanus sativus (laifuzi)	P284	Raphanus raphanistrum subsp. sativus (L.) Domin	Li et al. (2024c)
Salidroside	C14H_20_O_7_	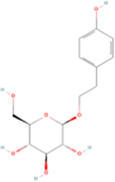	Rhodiola rosea (hongjingtian)	P161	Rhodiola rosea L.	Yang et al. (2022)

**TABLE 2:** Classification of herbal monomers and related parent structures.

Main contents	Sort	Representation of compounds	Molecular formula	Structure	References
Fisetin, Imperatorin, Tectorigenin, Formononetin, Kaempferitrin, Vitexin, Baicalein, Luteolin, Glabridin, Puerarin, Calycosin, Nobiletin Total flavones of Abelmoschus	Flavonoids	Flavone	C15H_10_O_2_	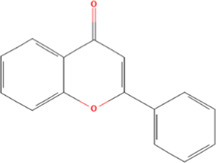	Li et al. (2022), Wang et al. (2024b), Yang et al. (2024), Hou et al. (2024), Li et al. (2024b), Lo et al. (2022), Song et al. (2023), Zhu et al. (2023), Liang et al. (2024a), Ye et al. (2025), Tan et al. (2022), Wang et al. (2023), Huang et al. (2022)
Ginkgolide B, Hederagenin, Astragaloside IV	Terpenoids	Isoprene	C5H_8_	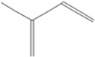	Qin et al. (2022), Chen et al. (2022), Jia et al. (2024)
Bergapten	Coumarins	Coumarin	C9H_6_O_2_	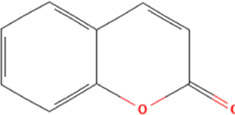	Li et al. (2025)
Schisandrin A	Lignans	Pinoresinol	C20H_22_O_6_	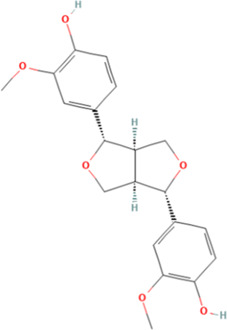	Wang et al. (2022b)
Rhein	Anthraquinones	Anthraquinone	C_14_H_8_O_2_	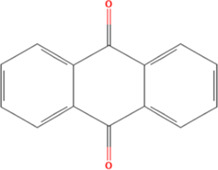	Xiong et al. (2023)
Radish red	Anthocyanins	Cyanidin	C15H_11_O_6_^+^	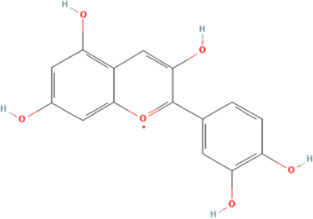	Li et al. (2024c)
Salidroside	Phenolic Glycosides	Salicin	C13H_18_O_7_	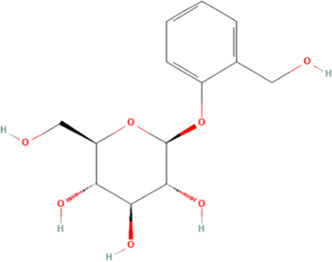	Yang et al. (2022)

**TABLE 3:** Evidence of monomers in treatment of renal fibrosis targeting ferroptosis.

Name	Experimental dose and models	Effect for observed biomarkers	Molecular mechanism	Effert for renal function	Effert for fibrotic marker	Detection of ferroptosis mitochondrial morphology	Ref.
Fisetin	*In vivo*: adenine diet- and UUO mice; Dose: 50 or 100 mg/kg/d *In vitro*: adenine or TGF-β1 treated TCMK-1; Dose: 20 μM	Fe2+ (↓), ACSL4 (↓), MDA (↓), COX2 (↓), GPX4 (↑), IL-1β (↓), IL-6 (↓), TNF-α (↓), MCP-1 (↓)	Decreased ACSL4 and COX2, increased GPX4	Scr (↓), BUN (↓), ACR (↓)	α-SMA (↓), FN (↓), Col-I (↓), Col-IV (↓), Col-VI (↓)	√	Wang et al. (2024b)
Imperatorin	*In vivo*: UUO mice; Dose: 30 mg/kg *In vitro*: Erastin treated NRK-52E; Dose: 0–100 μM	Fe2+ (↓), TFR1 (↓), ROS (↓), NOX-4 (↓), GPX4 (↑), SLC7A11 (↑)	Inhibition of oxidative stress and ferritin deposition	NA	α-SMA (↓), FN (↓)	NA	Yang et al. (2024)
Tectorigenin	*In vivo*: UUO mice; Dose: 100 mg/kg/d *In vitro*: erastin/RSL3 treated Mouse TECs; Dose: 20, 40,60 μM	ROS (↓), NOX4 (↓), 4-HNE (↓), p-Smad3 (↓), GPX4 (↑)	Inhibition of TGF-β1/Smad3/Nox4 aixs	Scr (↓), BUN (↓)	α-SMA (↓), FN (↓)	NA	Li et al. (2022)
Formononetin	*In vivo*: UUO mice and FA mice; Dose: 40 mg/kg/d *In vitro*: (pTECs); Dose: 0–100 μM	4-HNE (↓), GPX4 (↑), SLC7A11 (↑), Nrf2 (↑)	Suppressing the Smad3/ATF3/SLC7A11 pathway	Scr (↓), BUN (↓)	Col-I (↓), FN (↓), α-SMA (↓)	NA	Zhu et al. (2023)
Kaempferitrin	*In vivo*: UUO mice; Dose: 20 mg/kg/d *In vitro*: TECs; Dose: 20 μM, 40 μM, 80 μM	Fe2+ (↓), ROS (↓), 4-HNE (↓), MDA (↓), NOX4 (↓), GPX4 (↑), SLC7A11 (↑), IL-1β (↓), TNF-α (↓)	Enhanced thermal stability of NOX4	Scr (↓), BUN (↓)	α-SMA (↓), FN (↓)	NA	Li et al. (2024b)
Vitexin	*In vivo*: UUO and UIR mice; Dose: 30 mg/kg/*In vitro*: HK2 cells and NRK49F; Dose: 100 µM	Fe2+ (↓), MDA (↓), 4-HNE (↓), ACSL4 (↓), NRF2 (↑), HO-1 (↑), GPX4 (↑), IL-1β (↓), IL-6 (↓), TNF-α (↓), MCP-1 (↓)	Regulating KEAP1/NRF2/HO-1 pathway	NA	Col-I (↓), α-SMA (↓)	√	(Song et al., 2023) (Zhang et al., 2023b)
	*In vivo*: rats; Dose: 10 or 20 mg/kg/d *In vitro*: HK2 cells; Dose: 10 μM, 20 μM	Fe2+ (↓), ROS(↓), MDA (↓), GSH (↑), GPX4 (↑), SLC7A11 (↑)	Regulating SLC7A11/GPX4 pathway	Scr (↓), BUN (↓), UACR (↓)	Col-I (↓)		
Bergapten	*In vivo*: UUO mice; Dose: 100 mg/kg *In vitro*: HK2 cells; Dose: 100 or 200 μM	Fe2+ (↓), FTH1 (↑), MDA (↓), ACSL4 (↓), ROS (↓), PI3K (↓), GPX4 (↑), SLC7A11 (↑)	Inhibiteing PI3K phosphorylation and restoring GPX4 expression	Scr (↓), BUN (↓), urinary protein excretion (↓)	α-SMA (↓), FN (↓)	√	Li et al. (2025)
Nobiletin	*In vivo*: UUO mice; Dose: 50 mg/kg/d	TFR1 (↓), Nox4 (↓), SOD2 (↑), GPX4 (↑), SLC7A11 (↑), TNF-α (↓), IL-6 (↓)	Antioxidant	NA	α-SMA (↓), Col-I (↓), FN (↓)	NA	Lo et al. (2022)
Baicalein	*In vivo*: UUO rat; Dose: 100 mg/kg *In vitro*: MPC-5 cells; Dose: 0.1, 0.3, 1.0 μM	Fe2+ (↓), FTH (↑), ROS (↓), MDA (↓), SLC7A11 (↑), GPX4 (↑)	Antioxidant	24h-urinary albumin excretion (↓), Scr (↓), BUN (↓)	α-SMA (↓)	NA	Liang et al. (2024a)
Luteolin	*In vivo*: glyoxylate mice; Dose: 50 or 100 mg/kg *In vitro*:HK-2 cells; Dose: 100 μmol/L	Fe2+ (↓), MDA (↓), NR4A1 (↓), SLC7A11 (↑), GPX4 (↑)	Regulating Nr4a1-Slc7a11-GPX4 pathway	Scr (↓), BUN (↓)	Col-I (↓), α-SMA (↓)	√	Ye et al. (2025)
Astragaloside IV	*In vivo*: rat; Dose: 10 mg/kg *In vitro*:HK-2 cells; Dose: 100 μM	Fe2+ (↓), DMT1 (↓), TFR1 (↓), FPN1 (↑), FTL (↓), ROS (↓), MDA (↓), Nrf2 (↑), GPX4 (↑), SLC7A11 (↑)	Activating Nrf2 pathway, restoring GPx4 and SLC7A11	Scr (↓), BUN (↓)	Collegen (↓) (Masson staining)	√	Qin et al. (2022)
Ginkgolide B	*In vivo*: C57BL/KsJdb/db mice; Dose:100 or 200 mg/kg *In vitro*: MPC5 cells; Dose: 20, 40, or 80 µM	Fe2+ (↓), TFR1 (↓), FTH1 (↑), GPX4 (↑)	Inhibiting ubiquitination of GPX4	BUN (↓), 24-h UAE (↓)	Col-I (↓), α-SMA (↓)	NA	Chen et al. (2022)
Glabridin	*In vivo*: rat; Dose: 50 mg/kg *In vitro*: NRK-52E cells;	Fe2+ (↓), TFR1 (↓), FTH1 (↑), ROS (↓), MDA (↓), GPX4 (↑), SLC7A11 (↑), SLC3A2 (↑)	Iron metabolism and Antioxidant	Scr (↓),BUN (↓), UER (↓)	Collegen (↓), (Sirius red staining)	NA	Tan et al. (2022)
Hederagenin	*In vivo*: C57BL/6J mice; Dose: 50 mg/kg/d *In vitro*: HK2 cells; Dose: 10, 20, and 40 μg/mL	ROS (↓), MDA (↓), NOX4 (↓), p-smad3 (↓), GPX4 (↑), SLC7A11 (↑)	Inhibition of NOX4/SLC7A11 pathway-mediated ferroptosis by regulation of Smad3	Scr (↓), BUN (↓), ACR (↓)	Col-I (↓), FN (↓), α-SMA (↓)	NA	Jia et al. (2024)
Puerarin	*In vivo*: SD rats; Dose: 100 mg/kg/d *In vitro*: GMCs cells; Dose: 1 μM and 10 μM	Fe2+ (↓), TF (↓), TFR1 (↓), DMT1 (↓), Cp (↓), FPN1 (↑), ACSL4 (↓), MDA (↓), GSH (↑), Gpx4 (↑)	Iron metabolism	Scr (↓), BUN (↓), UACR (↓)	Col-I (↓) Col-IV (↓), TGF-β(↓), α-SMA (↓)	NA	(Hou et al., 2024) (Jian et al., 2023)
	*In vivo*: I/R Rats; Dose: 50 or 100 mg/kg *In vitro*: H/R HK2 cells Dose: 1 μM and 10 μM	Fe2+ (↓), FSP1 (↑), MDA (↓), 4-HNE (↓), NOX4 (↓), ACSL4 (↓), GPX4 (↑)	Regulating TLR4/Nox4 pathway	NA	α-SMA (↓)		
Calycosin	*In vivo*: db/db mice; Dose: 10 or 20 mg/kg/d *In vitro*: HK2 cells; Dose: 0, 5, 10, 20, 40 and 80 μM	NCOA4 (↓), ROS (↓), MDA (↓), GSH (↑), GPX4 (↑)	Regulating GPX4	Scr (↓), BUN (↓)	Collegen (↓) (Masson staining)	NA	Huang et al. (2022)
Rhein	*In vivo*:C57BL/6J mice; Dose: 150 mg/kg/d *In vitro*: podocyte (MPC5) cells; Dose: 25 μg/mL	Fe2+ (↓), TFR1 (↓), ROS (↓), MDA (↓), NOX1 (↓), ACSL4 (↓), SLC7A11 (↑), GPX4 (↑)	Regulating of the Rac1/NOX1/-catenin axis	Scr (↓), BUN (↓), urinary albumin (↓)	α-SMA (↓)	NA	Xiong et al. (2023)
Total Flavones of Abelmoschus (TFA)	*In vivo*: rat; Dose: 136 mg/kg/d *In vitro*: NRK-52E cells; Dose: 20 μg/mL	Fe2+ (↓), TFR1 (↓), ROS (↓), ACSL4 (↓), GPX4 (↑), SLC7A11 (↑)	Iron metabolism and Antioxidant	Scr (↓), BUN (↓), UACR (↓)	Collegen (↓) (Masson staining)	√	Wang et al. (2023)
Schisandrin A	*In vivo*:C57BL/6 mice; Dose: 5/50/100 mg/kg *In vitro*: HRGECs; Dose: 25/50/100 μM	Fe2+ (↓), MDA (↓), ROS (↓), AdipoR1 (↑), AMPK (↑), Nrf2 (↑), HO-1 (↑), GPX4 (↑), IL-6 (↓), INF-γ (↓), TNF-α (↓), IL-1β (↓)	Regulating AdipoR1/AMPK/Nrf2/HO-1/GPX4 aixs	Scr (↓), BUN (↓), urinary albumin (↓)	Col-IV (↓)	√	Wang et al. (2022b)
Radish red	*In vivo*: mice; Dose: 400 mg/kg *In vitro*: HK2 cells; Dose: 200, 400, 600 μg/mL	Fe2+ (↓), ROS (↓), MDA (↓), 4-HNE (↓), NOX4 (↓), ACSL4 (↓), Nrf2 (↑), HO-1 (↑) SLC7A11 (↑), GPX4 (↑), IL-1β(↓), IL-6 (↓), IL-18 (↓), TNF-α (↓)	Regulating Nrf2 related pathway	Scr (↓), BUN (↓), UACR (↓)	Collegen (↓) (Sirius red staining)	NA	Li et al. (2024c)
Salidroside	*In vivo*: SAMP8 mice; Dose: 60 mg/kg/d	TFR1 (↓), FPN1 (↑), FTH1 (↑), MDA (↓), SLC7A11 (↑), GPX4 (↑)	Iron metabolism	Scr (↓), BUN (↓)	α-SMA (↓)	NA	Yang et al. (2022)

### 4.1 Flavonoids

Fisetin, a compound extracted from the traditional Chinese medicine Sophora flavescens (Kushen), and imperatorin (IMP), a compound derived from Angelica dahurica (Baizhi), exhibit significant nephroprotective effects and share common therapeutic properties, such as anti-inflammatory and antioxidant activities. Wang B. et al. (2024) studied the effects of fisetin on ferroptosis and renal fibrosis in CKD models, induced by adenine diets and unilateral ureteral obstruction (UUO). Their findings revealed that fisetin reduced serum creatinine (Scr), blood urea nitrogen (BUN), and urinary albumin-to-creatinine ratio (ACR). Furthermore, fisetin downregulated renal fibrosis markers, such as α-SMA, FN, Col-I and VI, and inflammatory cytokines including IL-1β, IL-6, TNF-α, and MCP-1. It also upregulated glutathione levels and the GSH/GSSG ratio while decreasing MDA levels, suggesting its protective role against oxidative stress. ACSL4 overexpression attenuated the protective effects of fisetin, indicating its potential to inhibit ferroptosis in kidney diseases. Similarly, treatment with IMP reversed the increases in α-SMA, FN, and TGF-β1 expression, restored E-cadherin expression, and reduced inflammatory infiltration. Additionally, IMP mitigated oxidative stress by restoring GPX4 and SLC7A11 expression, which were downregulated in the UUO model. IMP also effectively reversed erastin-induced ferroptosis by normalizing intracellular Fe^2+^ and ROS levels, emphasizing its ability to maintain redox homeostasis (Yang et al., 2024).

Both fisetin and IMP exhibited promising nephroprotective effects by modulating oxidative stress and ferroptosis. Their ability to regulate inflammatory responses and key signaling pathways make them potential therapeutic agents for treating renal fibrosis.

Puerarin (PUR), an isoflavone primarily extracted from the traditional Chinese medicinal herb Pueraria lobata (Ge Gen), exhibits a wide range of pharmacological effects. For instance, it improves cardiovascular and cerebrovascular function, prevents osteoporosis, offers hepatoprotection and neuroprotection, and possesses hypoglycemic properties. Hou et al. (2024) investigated the therapeutic effects of puerarin on diabetic nephropathy. Puerarin reduced the levels of ACSL4, LDH, and MDA while upregulating GSH and GPX4, suggesting its protective role against HG-induced oxidative stress. These findings indicated that puerarin mitigated ferroptosis by modulating iron homeostasis and redox balance, thereby inhibiting ECM accumulation and decelerating the progression of diabetic nephropathy-associated renal fibrosis. In a separate study, puerarin attenuated renal fibrosis, and suppressed the expression of Toll-like receptor 4 (TLR4) and NADPH oxidase 4 (NOX4), highlighting its potential to ameliorate I/R-induced ferroptosis and renal injury (Jian et al., 2023).

Tectorigenin (Tec), a bioactive isoflavone extracted from the traditional Chinese medicinal herb Belamcanda chinensis (She Gan), exhibits a wide range of pharmacological activities, including antibacterial, antiviral, antitussive, expectorant, anti-inflammatory, antioxidant, immunomodulatory, and antitumor properties. Tec significantly ameliorated renal dysfunction and fibrosis, evidenced by decreased levels of Scr, BUN, and kidney injury molecule-1 (KIM-1). Tec also downregulated collagen deposition and fibrotic markers, inhibited Smad3 phosphorylation and NOX4 expression, and restored GPX4 levels. These results suggested that Tec can protect the kidneys by inhibiting ferroptosis and modulating oxidative stress and the TGF-β1/Smad3 pathways (Li et al., 2022). Additionally, Kaempferitrin (KM) from Astragalus membranaceus was studied in a UUO mouse model, significantly lowering Fe^2+^, 4-HNE, MDA, and NOX4 levels in the kidney and increasing GSH, GPX4, and SLC7A11 expression (Li J. et al., 2024). This study suggests that KM ameliorates renal fibrosis by inhibiting NOX4-mediated ferroptosis in renal tubular cells. Besides, nobiletin (Nob) is a polymethoxyflavone derived from the TCM Citrus reticulata (Chenpi), which attenuated collagen deposition, inflammation, and renal ferroptosis by downregulating NOX4 expression in mice with UUO (Lo et al., 2022).

Vitexin and formononetin are phytochemicals derived from *Nelumbo nucifera* (lianzixin) and Astragalus membranaceus (Huangqi), respectively. Both of them demonstrated significant nephroprotective properties. Vitexin has been widely recognized for its diverse pharmacological activities, including antiarrhythmic, antihypertensive, anti-inflammatory, antioxidant, sedative, and hypoglycemic effects. Treatment with vitexin (30 mg/kg/day) significantly alleviated renal tubular injury and renal fibrosis and downregulated pro-inflammatory cytokines (IL-1β, IL-6, TNF-α, and MCP-1) in mice with UUO. Mechanistically, vitexin enhanced the nuclear translocation of NRF2 and increased HO-1 mRNA expression by binding to KEAP1, thereby activating the KEAP1/NRF2/HO-1 signaling pathway (Song et al., 2023). Furthermore, vitexin inhibited EMT, preserved mitochondrial integrity, and exhibited no adverse effects in ischemia-reperfusion injury and diabetic nephropathy (Song et al., 2023; Zhang S. et al., 2023). Notably, the nephroprotective effects of vitexin were weakened in NRF2-knockout mice, suggesting that vitexin inhibited ferroptosis by suppressing KEAP1-induced NRF2 degradation, thereby upregulatign GPX4 expression and suppressing lipid peroxidation.

Formononetin exerts renoprotective effects primarily by modulating the Nrf2/ARE and TGF-β1/Smad3 signaling pathways. Zhu et al. (2023) reported that formononetin attenuated UUO-induced renal injury by downregulating Fe^2+^ and 4-HNE levels, while upregulating SLC7A11 and GPX4 expression, demonstrating superior efficacy compared to valsartan. Furthermore, it inhibited the translocation of Smad3 and ATF3 in renal tubular epithelial cells treated with inducers of ferroptosis (RSL3 and erastin). Although Nrf2 expression was significantly reduced after exposure to RSL3 or erastin, formononetin restored the nuclear translocation of NRF2 in a dose-dependent manner.

Baicalein, a flavonoid extracted from Scutellaria baicalensis (Huangqin), exhibited significant therapeutic potential in the treatment of kidney diseases. Treatment with baicalein markedly attenuated renal injury and fibrosis in the mouse models of UUO. It reduced tubular capillary dilation, decreased interstitial space expansion, diminished inflammatory cell infiltration, alleviated perivascular exudation, and downregulated α-SMA expression. Furthermore, treatment with baicalein reduced the number of iron-positive cells and decreased ROS and MDA levels, while elevating the activity of antioxidant enzymes, such as SOD, glutathione, and GSH. These protective effects are mediated partly by downregulating TGF-β1 and Smad2 expression (Liang GQ. et al., 2024).

Luteolin (Lut), a natural flavonoid derived from *Lonicera japonica* (Jinyinhua), possesses diverse pharmacological properties, including anti-inflammatory, antimicrobial, antioxidant, hypolipidemic, hypoglycemic, and immunomodulatory properties. Luteolin demonstrated renoprotective effects against calcium oxalate (CaOx)-induced renal injury and fibrosis by modulating oxidative stress and ferroptosis-related pathways. Specifically, it regulates the intracellular levels of GSH, MDA, and Fe^2+^, and restores the expression of key ferroptosis-associated proteins, such as SLC7A11 and GPX4. These mechanisms may be regulated by the orphan nuclear receptor NR4A1, suggesting the involvement of a gene-mediated pathway in the anti-fibrotic effects of luteolin (Ye et al., 2025).

Glabridin (Glab), derived from Glycyrrhiza uralensis (Gan cao), and total flavones of Abelmoschus manihot (TFA) were shown to significantly improve the management of diabetes and inhibit ferroptosis. Both compounds ameliorated renal fibrosis and suppressed ferroptosis by downregulation Fe^2+^, TFR1, and ROS levels, while concurrently upregulating the decreased levels of SLC7A11 and GPX4 (Tan et al., 2022) (Wang et al., 2023). Although Glab and TFA both regulate iron metabolism and suppress oxidative stress to inhibit ferroptosis-mediated fibrogenesis, their distinct molecular mechanisms remain incompletely characterized. Further studies are warranted to elucidate their precise mode of action and validate their translational potential in clinical practice.

Calycosin (Cal), derived from Astragalus membranaceus, protects against renal injury in diabetic nephropathy. It reverses the decrease in GPX4 expression and the increase in NCOA4 expression (Huang et al., 2022). This finding indicates that Cal may ameliorate diabetic nephropathy by regulating GPX4 and NCOA4 levels.

### 4.2 Terpenoids

Astragaloside IV (ASIV), extracted from Astragalus membranaceus (Huang Qi), has been demonstrated to mitigate Adriamycin (ADR)-induced renal damage. ASIV reduces reactive oxygen species and malondialdehyde levels, while enhancing antioxidant enzymes like GPX and SOD and reducing iron accumulation in kidneys by regulating FPN1, DMT1, TFR1, and FTL. Additionally, ASIV improves mitochondrial structure, increases oxidative stress proteins GPX4, Nrf2, and HO-1, and activates the PI3K/Akt pathway for protection against ADR-induced oxidative stress (Qin et al., 2022). Ginkgo biloba (GB), derived from Ginkgo biloba (Yin Xingye), exhibits pharmacological effects like vasodilation and antioxidant properties. Chen et al. (2022) showed that GB alleviates diabetic nephropathy in mice by reducing BUN and urinary albumin, thereby improving renal function. GB also mitigates renal histological damage by inhibiting GPX4 ubiquitination and lowering iron and ROS levels. HDG, extracted from Acanthopanax senticosus (Ciwujia), protects against ferroptosis and renal fibrosis by inhibiting NOX4 and p-Smad3 in streptozotocin (STZ)-induced diabetic nephropathy (DN) model (Jia et al., 2024).

### 4.3 Coumarins

Bergapten, a natural coumarin derivative, has emerged as a promising therapeutic agent for renal fibrosis. It modulates the expression of GPX4. Studies have indicated that treatment with bergapten can indirectly restore GPX4 levels, thus inhibiting lipid peroxidation and mitigating the progression of renal fibrosis. Bergapten exerts its protective effects by inhibiting the phosphoinositide 3-kinase (PI3K) pathway, which regulates GPX4 expression. By inhibiting PI3K phosphorylation, bergapten upregulates GPX4 expression and enhances the antioxidant defense system in renal tubular epithelial cells. This restoration of GPX4 protects against ferroptosis and downregulates fibrotic markers, such as α-SMA and FN, in the kidney (Li et al., 2025).

### 4.4 Lignans

Schisandrin A (Sch A) is a prominent lignan compound primarily extracted from the fruit of Schisandra chinensis. Sch A has emerged as a promising therapeutic agent for renal fibrosis. Particularly, it can regulate iron metabolism-related proteins. Sch A can downregulate key ferroptosis markers, such as GPX4 and SLC7A11, suggesting its role in preserving cellular integrity and function in stress conditions. Furthermore, activation of the Nrf2 signaling pathway by Sch A may contribute to its antioxidative effects, enhancing the resilience of the kidney against oxidative damage and subsequent fibrosis (Wang X. et al., 2022). Overall, this study underscores the potential of Sch A in treating renal fibrosis by inhibiting ferroptosis and oxidative stress, paving the way for future clinical applications.

### 4.5 Anthraquinones

Rhein is derived from Rheum palmatum (Da Huang), which possesses purgative, anti-inflammatory, anti-tumor, hepatoprotective, and lipid-lowering properties, improves vascular function, and preserves renal function. Recent studies have elucidated the mechanisms by which Rhein exerts its protective effects, notably through Rac1 inhibition. Rhein was shown to downregulate Rac1 expression, which correlates with decreased NOX1-mediated ROS production and downregulation of fibrosis markers, such as α-SMA and TGF-β (Xiong et al., 2023). Furthermore, inhibition of Rac1 activity by Rhein not only reduces oxidative stress but also affects downstream signaling pathways involved in EMT and renal fibrosis, such as the β-catenin pathway (Xiong et al., 2023). This finding suggests the ability of Rhein to inhibit Rac1, which finally improves renal fibrosis.

### 4.6 Other monomers

Radish red (RR) is an anthocyanin extracted from the red-fleshed root of Raphanus sativus L., chemically identified as pelargonidin-3-sophoroside-5-glucoside. RR possesses significant potential in the development of natural food colorants, functional antioxidants, and nutritionally fortified foods. The dried mature seeds of Raphanus sativus L., known as Raphanus sativus (Laifuzi), possess medicinal properties, promote digestion, relieve bloating, descend qi, and resolve phlegm. In the mice model of CKD, RR reduced the levels of LDH and kidney injury markers, including KIM-1 and neutrophil gelatinase-associated lipocalin (NGAL). RR exerted its protective effects by activating the Nrf2 signaling pathway (Li Q. et al., 2024).

Salidroside (SAL), a phenylpropanoid glycoside, is primarily derived from the roots of various species of the Rhodiola genus, particularly Rhodiola rosea. This compound has garnered significant attention due to its diverse biological activities, which include antioxidant, anti-inflammatory, neuroprotective, and cardioprotective properties (Liang K. et al., 2024). SAL has emerged as a potential therapeutic agent in mitigating renal fibrosis, primarily through its ability to modulate iron metabolism. Studies have indicated that SAL can effectively regulate the expression of key iron transport proteins, including DMT1 and FPN1, which are crucial for iron uptake and export, respectively. Treatment with SAL significantly decreased iron accumulation in renal tissues, which was associated with reduced levels of TFR1 and increased ferritin expression, thereby inhibiting ferroptosis and fibrosis (Yang et al., 2022).

## 5 Clinical relevance and translational potential

Currently, the treatment of CKD is still based on controlling the etiology, such as poorly controlled diabetes mellitus (DM), hypertension, glomerular disease, and glomerulonephritis (Webster et al., 2017). The international standard of care for slowing the progression of CKD recommends the blockade of the renin-angiotensin-aldosterone system (RAAS) with angiotensin-converting enzyme inhibitors, angiotensin II receptor type 1 (AT1) antagonists, or direct renin blockers. However, RAAS blockade is not sufficient to halt the progression of CKD and renal fibrosis (Klinkhammer et al., 2017).

Great progress has been made in the prevention and treatment of renal fibrosis by regulating ferroptosis using CHMs. Compared to RAAS inhibitors (candesartan, irbesartan, and valsartan), CHMs (IMP (Yang et al., 2024), Tec (Li et al., 2022), formononetin (Zhu et al., 2023), and Nob (Lo et al., 2022)) have a comparable effect in ameliorating renal fibrosis in UUO kidney.

Astragalus membranaceus (Huang Qi) is one of the most widely used traditional CHMs. It has been used in treatment of various kidney diseases for many years (Fu et al., 2014). Randomized controlled trials have demonstrated that compared to monotherapy with RAAS inhibitors alone, Astragalus membranaceus combined with RAAS inhibitors can offer superior efficacy in the clinical treatment of DN (Lin et al., 2024). Four monomers from Astragalus membranaceus, including formononetin (Zhu et al., 2023), kaempferitrin (Li J. et al., 2024), calycosin (Huang et al., 2022) and astragaloside IV (Qin et al., 2022), markedly ameliorated renal fibrosis and ferroptosis similar to the ferroptosis inhibitor Fer-1. These findings suggest that monomers of Astragalus have great potential for clinical applications.

Several studies have reported the protective effects of Abelmoschus manihot in patients with CKD, without any noticeable side effects (Zhao et al., 2022; Zhang et al., 2014; Li P. et al., 2020). TFA, a bioactive phytochemical complex derived from the Abelmoschus manihot, exerts significant anti-fibrotic activity in the experimental models of renal diseases. Wang et al. (2023) reported that TFA can attenuate renal fibrosis similar to dapagliflozin, an established first-line medication for CKD (Heerspink et al., 2020; KDIGO, 2024, 2024). Collectively, these findings position TFA as a promising therapeutic candidate for targeted anti-fibrotic therapy in progressive renal diseases.

Currently, there are no specific drugs for renal fibrosis. Monomers of CHMs possess multiple effects, multiple targets, and strong activity. They have become a recent focus of research and drug development (Song et al., 2024). However, research on the regulation of ferroptosis in renal fibrosis by CHMs is still in the preliminary stage. Future studies should explore the combination of monomers of CHMs to discover new drugs for the treatment of renal fibrosis.

## 6 Perspectives and conclusion

This review showed that CHM monomers can regulate iron homeostasis, inhibit lipid peroxidation, balance oxidative stress, and modulate EMT through their effects on relevant signaling pathways and molecular targets, thereby reducing Scr and BUN levels, mitigating proteinuria, protecting against pathological fibrosis, suppressing the inflammatory response, and reducing the expression of Col and FN in the kidneys. They can delay or reverse fibrosis in various animal models and cell models of CKD. Most monomers of CHMs can reduce Fe^2+^ load, possibly by improving iron transport through the regulation of TF, TFR, DMT1, and FPN1. They maintain iron reserves by stabilizing FTH1 and FTL and inhibit ferritinophagy by acting on NCOA4 and Nrf2, thereby maintaining iron homeostasis and inhibiting ferroptosis. Among them, IMP, GB, TFA, and RR can markedly reduce Fe^2+^ levels. Cal can inhibit NCOA4, thereby suppressing ferritinophagy and reducing Fe^2+^ levels. Fisetin, vitexin, Rhein, TFA, and RR can inhibit the target ACSL4, thereby improving lipid peroxidation. IMP, Tec, KM, Nob, HDG, RR, and PUR can significantly inhibit NOX4. This study found that many CHM monomers can upregulate GPX4 levels, and PUR can increase FSP1 levels. This study also found that FN, vitexin, Nob, ASIV, schisandrin A, and RR can increase Nrf2 levels and inhibit ferroptosis through several pathways. Additionally, Tec, FN, and HDG can regulate ferroptosis and fibrosis through Smad3. The results showed that FN is more effective than valsartan. The effects of ASIV and DFO were shown to be similar, and the effects of TFA were shown to be like those of dapagliflozin. These results emphasize the potential of CHM monomers in providing clinical benefits.

Most monomers mentioned in this study were flavonoids, which can strongly suppress ferroptosis and renal fibrosis. Flavonoids, such as formononetin, kaempferitrin, astragaloside IV, and calycosin, all are derived from Astragalus membranaceus, which can modulate Fe^2+^, Smad3/ATF3/SLC7A11, NOX4, Nrf2, GPX4, and NCOA4, lower Scr and BUN levels. We look forward to their combined use to enhance their efficacy for renal fibrosis.

In addition to deepening research on current hotspots, such as GPX4 and Nrf2, it is essential to investigate the monomers of CHMs that target key nodes in iron metabolism. These nodes include ferritin autophagy mediated by NCOA4, the lipid remodeling enzyme system ACSL4, and the novel antioxidant axis involving FSP1-CoQ10. Further studies on the exact mechanisms by which these monomers affect ferroptosis and fibrosis are necessary to promote their clinical application.

Some challenges faced by CHM monomers include poor water solubility, low bioavailability, and inadequate stability. Monomers like, bergapten and tectorigenin, exhibit low bioavailability due to poor aqueous solubility and rapid metabolism. For example, only high doses (50 mg/kg in UUO mice) of kaempferitrin can achieve therapeutic effects (Li J. et al., 2024), which may not be feasible in humans without optimized formulation. Although astragaloside IV is linked to Nrf2 activation (Qin et al., 2022), its specific targets in iron metabolism (e.g., hepcidin or transferrin receptor regulation) remain uncharacterized. Similarly, direct evidence linking schisandrin A, rhein, and their antioxidant properties to ferroptosis modulation in renal cells are lacking. Furthermore, no clinical trials have evaluated these monomers for renal fibrosis. Preclinical models (UUO or 5/6 nephrectomy) do not fully recapitulate human CKD heterogeneity, such as comorbidities (diabetes, hypertension) or drug-drug interactions.

In summary, CHM monomers possess significant potential for treating renal fibrosis. Advancements in technical methods can help develop more effective, targeted, and safe treatment options to address the challenges in the treatment of renal fibrosis. Future large-sized randomized controlled trials are needed to verify the efficacy and safety of CHM monomers, offering new hope for the treatment of CKD and renal fibrosis.

This review provides a comprehensive overview of monomer research methods and identifies key targets for regulating ferroptosis. The 21 CHM monomers can regulate iron homeostasis, inhibit lipid peroxidation, and regulate oxidative stress and EMT by affecting relevant signaling pathways and molecular targets, thereby delaying or reversing fibrosis. The majority of the herbal monomers mentioned in this study belong to flavonoids, which have strong potential in treating CKD and renal fibrosis.

## Glossary

CKD: chronic kidney disease
CHMs: Chinese herbal medicines
Fer-1: ferrostatin-1
Lip-1: liproxstatin-1
EMT: epithelial-mesenchymal transition
TGF-β1: Transforming growth factor-β1
TGF-βRI: TGF-β receptor I
TGF-βRII: TGF-β receptor II
TEC: tubular epithelial cells
Col-I: collagen I
TFR1: transferrin receptor 1
FPN1: ferroportin 1
STEAP3: Six-transmembrane epithelial antigen of the prostate 3
DMT1: Divalent metal transporter 1
LIP: labile iron pool
FTH1: ferritin heavy chain 1
FTL: ferritin light chain
NCOA4: Nuclear receptor coactivator 4
Nrf2: Nuclear factor erythroid 2-related factor 2
ACC: acetyl-CoA carboxylase
ACSL4: Acyl-CoA synthetase long-chain family member 4
AA: arachidonic acid
AdA: adrenic acid
PE: phosphatidylethanolamine
LPCAT3: lysophosphatidylcholine acyltransferase 3
NOXs: NADPH oxidases
LOXs: lipoxygenases
COXs: cyclooxygenases
PUFA-OOH: production of lipid hydroperoxides
HO-1: heme oxygenase-1
AMPK: AMP-activated protein kinase
MDA: malondialdehyde
4- HNE: 4-hydroxynonenal
GPX4: glutathione peroxidase 4
GSH: glutathione
ATF3: activating transcription factor 3
NADPH: nicotinamide adenine dinucleotide phosphate
FSP1: ferroptosis suppressor protein 1
CoQ10: coenzyme Q10
ARE: antioxidant response element
PT: proximal tubule
FTH: ferritin
MCP-1: monocyte chemoattractant protein-1
IMP: imperatorin
UUO: unilateral ureteral obstruction
Scr: serum creatinine
BUN: blood urea nitrogen
ACR: albumin-to-creatinine ratio
PUR: Puerarin
TLR4: Toll-like receptor 4
NOX4: NADPH oxidase 4
Tec: Tectorigenin
KIM-1: kidney injury molecule-1
Nob: nobiletin
Glab: Glabridin
TFA: total flavones of Abelmoschus manihot
ASIV: Astragaloside IV
GB: Ginkgo biloba
STZ: streptozotocin
DN: diabetic nephropathy
PI3K: phosphoinositide 3-kinase
Sch A: Schisandrin A
SAL: Salidroside
DM: diabetes mellitus
RAAS: renin-angiotensin-aldosterone system
AT1: angiotensin II receptor type 1
FN: fibronectin
SLC7A11: solute carrier family 7 member 11
SLC3A2: solute carrier family 3 member 2
FAO: fatty acid oxidation
ATP: adenosine triphosphate
